# Vertical Root Fracture initiation in curved roots after root canal preparation: A dentinal micro-crack analysis with LED transillumination

**DOI:** 10.4317/jced.54227

**Published:** 2017-10-01

**Authors:** Ramón Miguéns-Vila, Benjamín Martín-Biedma, Purificación Varela-Patiño, Manuel Ruíz-Piñón, Pablo Castelo-Baz

**Affiliations:** 1 DDS, University of Santiago de Compostela; Master of Endodontics, Entrerrios Street, no number, 15702, Santiago de Compostela; 2 PhD, University of Santiago de Compostela; Master of Endodontics, Entrerrios Street, no number, 15702, Santiago de Compostela; 3 DDS, PhD, University of Santiago de Compostela; Master of Endodontics, Entrerrios Street, no number, 15702, Santiago de Compostela

## Abstract

**Background:**

One of the causative factors of root defects is the increased friction produced by rotary instrumentation. A high canal curvature may increase stress, making the tooth more susceptible to dentinal cracks. The purpose of this study was to evaluate dentinal micro-crack formation with the ProTaper NEXT and ProTaper Universal systems using LED transillumination, and to analyze the micro-crack generated at the point of maximum canal curvature.

**Material and Methods:**

60 human mandibular premolars with curvatures between 30–49° and radii between 2–4 mm were used. The root canals were instrumented using the Protaper Universal® and Protaper NEXT® systems, with the aid of the Proglider® system. The obtained samples were sectioned transversely before subsequent analysis with LED transillumination at 2 mm and 8 mm from the apex and at the point of maximum canal curvature. Defects were scored: 0 for no defects; and 1 for micro-cracks.

**Results:**

Root defects were not observed in the control group. The ProTaper NEXT system caused fewer defects (16.7%) than the ProTaper Universal system (40%) (*P*<0.05). The ProTaper Universal system caused significantly more micro-cracks at the point of maximum canal curvature than the ProTaper NEXT system (*P*<0.05).

**Conclusions:**

Rotary instrumentation systems often generate root defects, but the ProTaper NEXT system generated fewer dentinal defects than the ProTaper Universal system. A higher prevalence of defects was found at the point of maximum curvature in the ProTaper Universal group.

** Key words:**Curved root, Micro-crack, point of maximum canal curvature, ProTaper NEXT, ProTaper Universal, Vertical root fracture.

## Introduction

Vertical root fractures are a complication that can be commonly found on endodontically treated teeth (1). This complication often leads to teeth extraction or root amputation (2), therefore, we must avoid the propagation of dentinal defects such as micro-cracks that can be related to root fracture initiation.

Endodontic biomechanical preparation techniques can damage the root dentin since the larger instrument’s taper may eliminate a greater amount of root dentin, elevating the level of stress (3) of the instrument as well as the number of rotations of the file inside the canal. These factors can increase the occurrence of root defects such as micro-cracks.

Dentinal micro-cracks are a clinical complication that can be generated during root canal procedures. Those defects can lead to root fracture, and must be prevented (4-7). Micro-cracks produced during shaping procedures can propagate by occlusal forces with repeated stress application and finally result in root fractures (8,9). Canal micro-cracks originate inside the root canal, and may or may not reach the external root surface (10). Previous studies have attributed root defects to root canal instrumentation (3,6), obturation procedures (6,11), a high concentration of sodium hypochlorite (12), complex dental anatomy (13), and retreatments (14). Canal shape seems to be an important factor, with a reduced radius of curvature strongly influencing stress concentration (15). A low radius of canal curvature can increase stress (15), which renders the root more susceptible to dentinal micro-cracks, and consequently, root fractures (16).

The introduction of the M-Wire alloy has permitted the development of new rotary instruments with improved mechanical properties that help to preserve root anatomy. The ProTaper NEXT files are composed of M-Wire nickel-titanium alloy. They have an off-centered rectangular design that minimizes contact between the file and the dentin, and their progressive and regressive percentage tapers allow the use of fewer instruments on the root canal preparation compared to the ProTaper Universal system. The ProTaper Universal files are composed of standard NiTi alloy. This system is characterized by an increasing taper design that removes relatively more dentin coronally compared to other systems (17).

Recent studies assessed dentinal crack generation with LED transillumination after root canal instrumentation with different systems (19).

The purpose of this study was to evaluate dentinal micro-crack formation with the ProTaper NEXT and ProTaper Universal systems using LED transillumination, and to analyze the micro-crack generated at the point of maximum canal curvature.

## Material and Methods

-Sample preparation

In total, 60 mandibular premolars with a root canal curvature between 30° and 49° and a radius between 2 mm and 4 mm (19), extracted for reasons not related to this study, were selected and stored in distilled water until use. The external root surface was inspected using a stereomicroscope (Leica MZ16F; Leica Microsystems Heidelberg GmbH, Mannheim, Germany) to exclude the possibility of any micro-cracks or defects before the procedure. Radiographs were taken from buccolingual and mesiodistal aspects to determine the root canal curvature at the point of maximum canal curvature according to Pruett’s method (19), which describes it as an angle measured in degrees and a radius measured in millimeters. The coronal portion of all teeth was removed using a low-speed saw (Isomet 4000; Buehler Ltd; Lake Bluff, IL) with water cooling, obtaining a standardized root length of 16 mm.

To create an artificial periodontal ligament, the root was covered with a single layer of aluminum foil, embedded in an acrylic tube filled with acrylic resin (Duralay Dental Mfg Co, Worth, IL), and removed after setting. The aluminum foil was removed from the root surface. A hydrophilic polyvinyl siloxane material (Elite HD+ Light Body Set: Zhermack Spa; Rovigo, Italy) replaced the space left by the foil, and the root was immediately repositioned (4).

-Shaping and cleaning

The teeth were randomly divided into three groups (n=20) before commencing instrumentation.

Group 1: All of the samples were preflared using a #10 K-Flexofile (Dentsply Maillefer; Ballaigues; Switzerland), followed by a Proglider® (Dentsply; Maillefer; Ballaigues; Switzerland) to create an appropriate glidepath. For instrumentation, the Protaper Universal® system and X-Smart® motor (Dentsply; Maillefer; Ballaigues; Switzerland) were used. The sequence indicated by the manufacturer was followed through to a final file diameter of 30 mm at its point (F3).

Group 2: All of the samples were preflared using a #10 K-Flexofile, followed by a Proglider® to create an appropriate glidepath. For instrumentation, the Protaper NEXT® system (Dentsply Maillefer; Ballaigues; Switzerland) and X-Smart® motor were used. The sequence indicated by the manufacturer was followed through to a final file diameter of 30 mm at its point (X3).

Group 3 (control group): Left unprepared. All teeth were irrigated with 2 mL NaOCl 5.25% (Dentaflux; J. Ripoll SL; Madrid, Spain) using a Max-I-Probe needle (Hawe Neos Dental SA; Bioggio, Switzerland). After preparing the canals, all of the specimens were irrigated with 3 mL NaOcl 5.25%, activating the irrigation with manual dynamic irrigation (20).

-Sectioning and microscopic evaluation

All of the roots were horizontally sectioned at 2 mm, 8 mm from the apex and at the point of maximum canal curvature (the point of maximum curvature for all of the samples was between 3 mm and 7 mm) using a low-speed saw (Isomet 4000) under water cooling. This sawing action does not cause any dentinal defects (21). We assigned the maximum canal curvature as the intersection of two perpendicular lines to the long axes of the coronal and apical portions of the root canal space as described by Pruett (19). Slices were then examined through a stereomicroscope (Leica MZ16F) with LED transillumination, and digital images were captured for subsequent analysis (Leica DFC490; Leica Microsystems Heidelberg GmbH, Mannheim, Germany). Each of the three levels were examined for defects and scored accordingly: 0 for no defects; and 1 for micro-cracks (a fissure extending from the root canal interior towards the external root surface) (8).

-Statistical analysis

The final result was obtained through statistical analysis of the scores obtained for each level studied in the two groups. Pearson’s chi-squared test was performed to compare the incidence of micro-cracks between the experimental groups. *P* values less than 0.05 were considered statistically significant. The SPSS software program (SPSS Inc, Chicago, IL, version 22) was used to perform statistical analyses.

## Results

**Figure 1:**
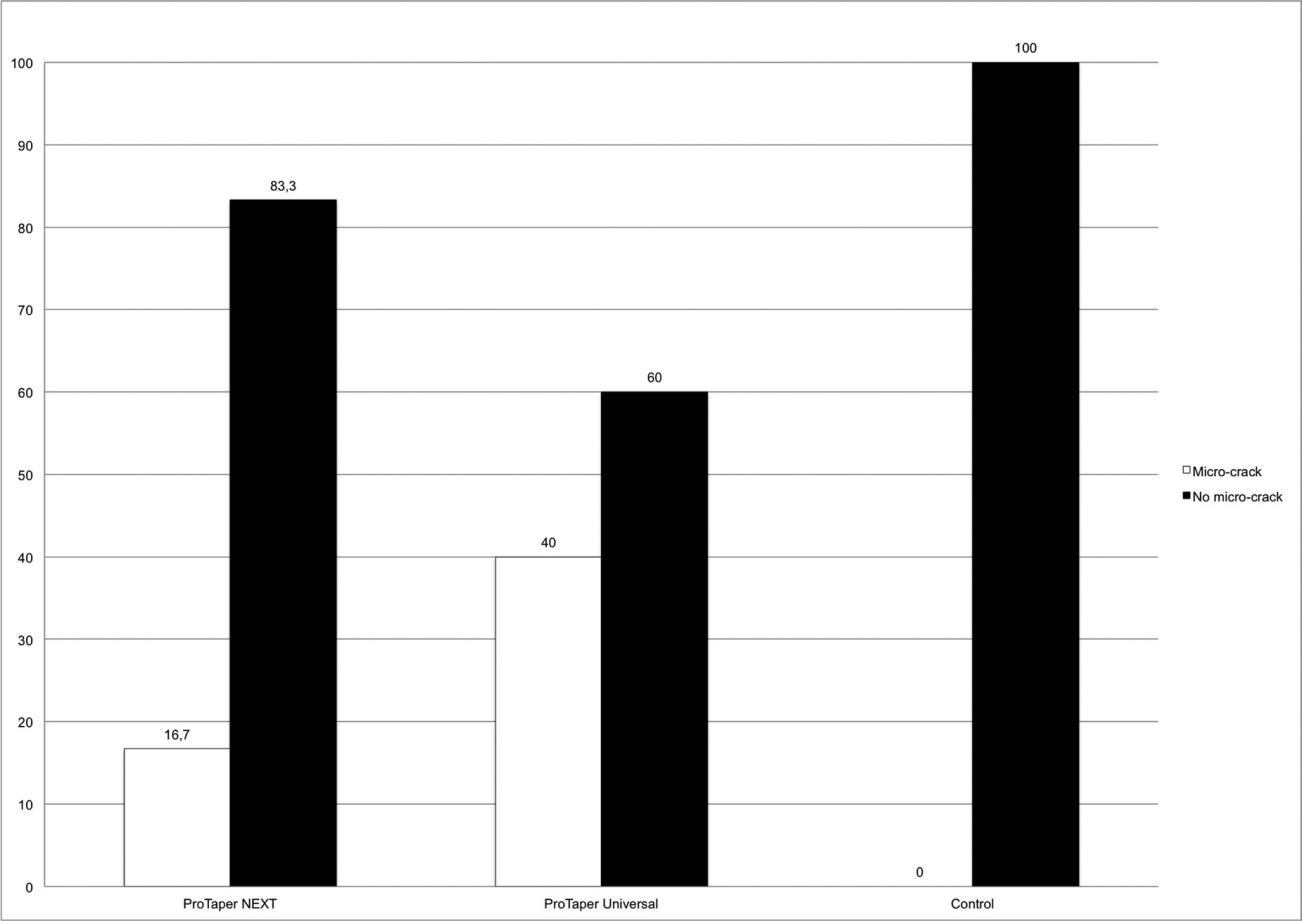
Percentage of micro-cracks in each study group. There was a statistically significant difference between the groups (*P*<0.01).

**Figure 2:**
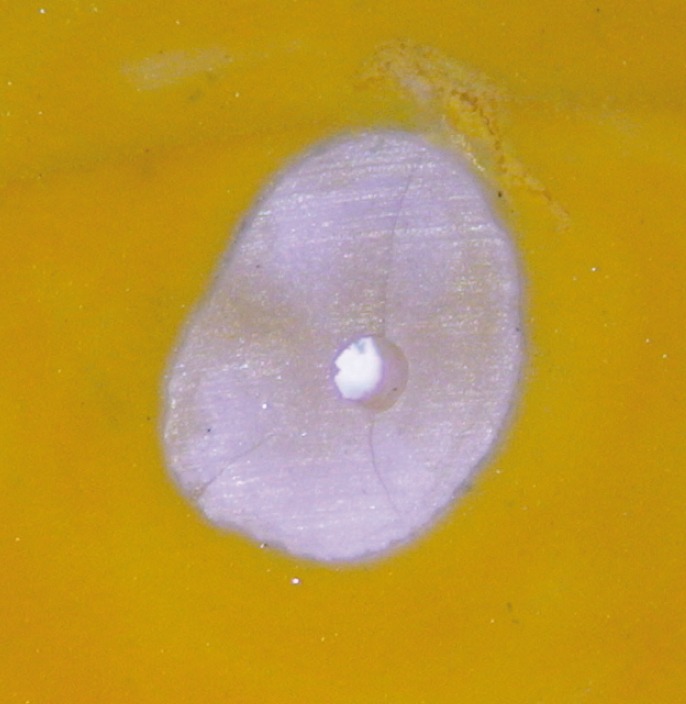
Sample of the ProTaper Universal group with dentinal cracks.

**Figure 3:**
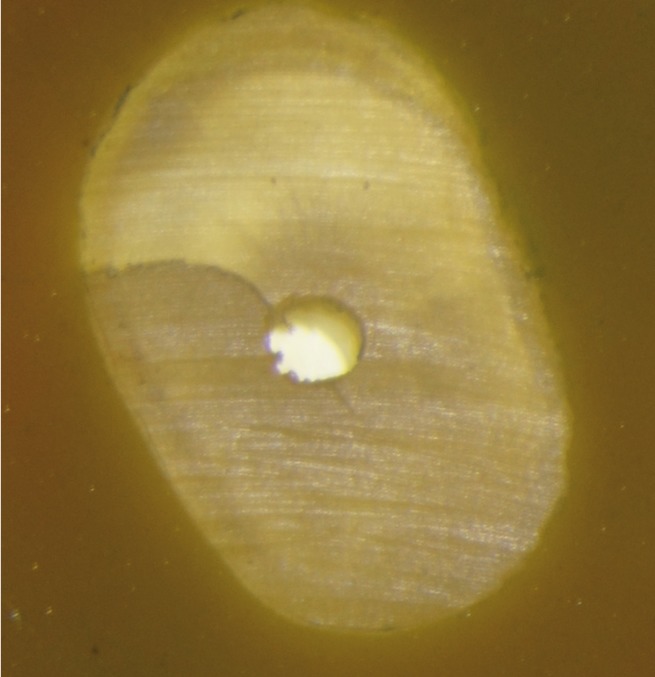
Sample of the ProTaper NEXT group with dentinal cracks.

 Table 1 and Figure 1 show the number and percentage of roots that developed micro-cracks (Figs. 2,3) in each group. Micro-cracks were not observed in the control group. The ProTaper Universal instruments caused more micro-cracks (40%) than the ProTaper NEXT instruments (16.7%) (*P*<0.05). There were statistically significant differences in micro-crack incidence on the examined areas when using the ProTaper NEXT system (*P*<0.05). No defects were found 2 mm from the apex (0%), 35% were found 8 mm from the apex, and fewer defects were found at the point of maximum canal curvature (15%). More defects were found at the point of maximum curvature using the ProTaper Universal, system and the difference was statistically significant (*P*<0.05).

**Table 1:**
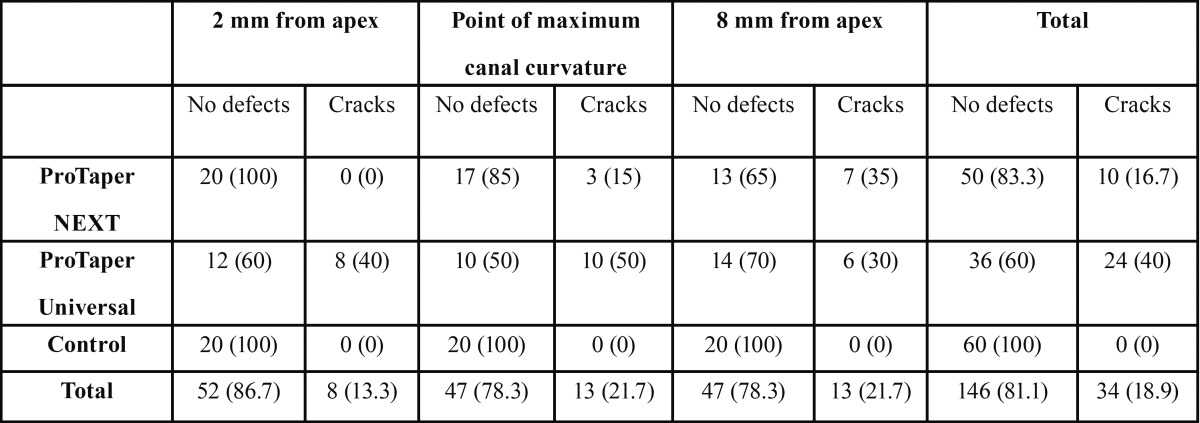
Number and percentage of micro-cracks after instrumentation with different systems (*P*<0.01). The ProTaper NEXT system produced significantly fewer micro-cracks as it approached the apex (*P*<0.05). At the point of maximum canal curvature, the ProTaper Universal system generated significantly more defects than the ProTaper Universal system (*P*<0.05).

## Discussion

Dentinal micro-cracks are a clinical situation that can evolve to root fractures, a complication that can cause the extraction of the tooth. It is essential for the preservation of the teeth to acknowledge which rotary instrumentation system is safer to use regarding dentinal micro-crack generation.

In the present study, ProTaper Universal files caused significantly more micro-cracks than ProTaper NEXT files. In all, 40% of the ProTaper Universal samples and 16.7% of the ProTaper NEXT root sections had dentinal micro-cracks after root canal instrumentation. By comparison, the negative control group showed no micro-cracks. Comparing our results with other studies conducted on straight canals, we found some similarities and differences. Capar *et al.* (22) investigated and compared dentinal micro-cracks generated after instrumentation with the ProTaper NEXT and ProTaper Universal systems. Micro-cracks were observed in 56% of the ProTaper Universal specimens and in 28% of the ProTaper NEXT samples, results that are consistent with our findings. Capar *et al.* (22) used exclusively mandibular premolars with a root canal curvature of less than 10°, and the rotary instrumentation systems were used until a final file diameter of 40 mm at its point was reached (F4 and X4). For this reason, it is possible that the incidences identified in their study were higher than in our results. Karataş *et al.* (23) also compared dentinal micro-crack generation with the same two systems, and found no statistically significant difference among the experimental groups. Dentinal micro-cracks were found in 33% of the ProTaper NEXT samples and in 37% of the ProTaper Universal specimens. Nevertheless, the ProTaper NEXT system produced significantly fewer micro-cracks than the ProTaper Universal in the apical section. These results on the apical section concur with the results of our study. Mandibular central incisors with straight root canals (<5°) were used by Karataş *et al.*, (23) and the samples were instrumented until a final file of diameter 25 was achieved at each point.

The structural configuration of the files (24) could contribute to the formation of dentinal micro-cracks. Liu *et al.* (25) studied the incidence of defects produced by the Self-Adjusting File (SAF). This file has a hollow thin-walled design that is compressible, and has an abrasive surface. The SAF can adapt its shape to the canal anatomy, and its vibrating movement with a continuous flow of irrigant facilitates its cleaning and minimizes friction (26,27) In the study by Liu *et al.* (25), the SAF instrument caused no micro-cracks at all. The authors analyzed microcrack generation after using three single-file systems, and compared the results with those of the ProTaper Universal system. All three single-file systems caused fewer root microcracks than the ProTaper Universal system. These results may be explained by the finding that more manipulations in the canal could cause accumulated damage (28). Accordingly, the number of instruments may influence micro-crack generation. In our study, the ProTaper NEXT system employed fewer instruments, which could be responsible for the lower quantity of defects in this group.

The composition of the file (29) is an important factor, given that the M-Wire alloy in the ProTaper NEXT system confers some improved mechanical properties to instruments, while concurrently helping to preserve the original anatomy of the root canal (30-32). The taper of the files may contribute to the formation of dentinal defects (33). Wilcox *et al.* (4) stated that there may be a greater risk of root fracture when more root dentin is removed. Hin *et al.* (34) compared the incidence of root micro-cracks after using SAF, ProTaper, and Mtwo. The present study attributed the higher incidence of micro-cracks observed in the ProTaper system to its larger taper. The SAF file has no taper, and caused the least amount of defects among the experimental groups. Therefore, many factors may influence dentinal micro-crack generation. Furthermore, most of the studies in the literature concur with our results that rotary instrumentation techniques may cause defects and debilitate the root (22,23,25,33-36).

Previous studies have analyzed micro-crack generation on straight canals; on the other hand, none have evaluated dentinal defect formation on curved root canals. Schäfer *et al.* (37) investigated the frequency and degree of canal curvatures; 84% of the root canals examined were curved and 24% of those were severely curved. Pineda *et al.* (38) found that only 3.1% of the roots were straight canals in their study. Lertchirakarn *et al.* (15) considered that the increased degree of curvature could raise the susceptibility to fractures, and accordingly, the possibility of dentinal micro-crack generation. Therefore, canal curvature may be an important factor of defect formation, with a strong generation of stress at the point of maximum canal curvature. Given those hypotheses, we studied micro-crack generation on curved roots, and found that there were no significant differences in micro-crack formation between the three sections of the ProTaper Universal group. In the ProTaper NEXT group, no micro-cracks were observed at 2 mm from the apex, only 15% were found in the point of maximum canal curvature, and similar results were obtained with the ProTaper Universal group in the coronal third. At the point of maximum curvature, there were statistically significant differences between the experimental groups, with fewer micro-cracks caused by the ProTaper NEXT system. This may be a result of the combination of a correct glidepath using the Proglider file and instrumentation with the ProTaper NEXT files. The Proglider file could generate a straightforward path with smooth walls toward the apex. Moreover, the taper of the files used and the M-Wire alloy of the Proglider and the ProTaper NEXT instruments may be responsible for the lower incidence of dentinal micro-cracks on curved roots. The Proglider instruments have enhanced mechanical properties, including higher flexibility than systems manufactured from other alloys (39). On the other hand, in the ProTaper Universal group, we found a higher generation of defects at the point of maximum curvature, likely due to the higher taper, alloy composition, and greater quantity of instruments used.

Within the limitations of this study, instrumentation of root canals with the ProTaper Universal and ProTaper NEXT system was found to damage root canal dentin. Rotary instrumentation with ProTaper NEXT generated fewer micro-cracks compared to the Protaper Universal system (*p*<0.05). With the ProTaper NEXT system, fewer dentinal micro-cracks formed at the apical third and at the point of maximum curvature than the coronal area, in roots with curvatures between 30° and 49° (*p*<0.05). At the point of maximum curvature, the ProTaper NEXT system caused significantly fewer micro-cracks than the ProTaper Universal instruments (*P*<0.05).

Therefore, based on our results, the use of ProTaper NEXT as instrumentation is safer regarding possible vertical root fracture propagation or initiation.
